# Unveiling the interoception impairment in various major depressive disorder stages

**DOI:** 10.1111/cns.14923

**Published:** 2024-08-18

**Authors:** Hongliang Zhou, Jikang Liu, Yuqing Wu, Zixuan Huang, Wenliang Wang, Yuhang Ma, Haohao Zhu, Zhenhe Zhou, Jun Wang, Chenguang Jiang

**Affiliations:** ^1^ Department of Psychology The Affiliated Hospital of Jiangnan University Wuxi China; ^2^ Department of Psychiatry The Affiliated Wuxi Mental Health Center of Nanjing Medical University Wuxi China; ^3^ Department of Psychiatry The Affiliated Mental Health Center of Jiangnan University Wuxi China; ^4^ Department of Psychosomatics and Psychiatry, ZhongDa Hospital, School of Medicine Southeast University Nanjing China

**Keywords:** interoception, MAIA‐2, major depressive disorder, pathophysiological mechanism, PHQ‐9, the preclinical phase of depression

## Abstract

**Background:**

The intricate pathophysiological mechanisms of major depressive disorder (MDD) necessitate the development of comprehensive early indicators that reflect the complex interplay of emotional, physical, and cognitive factors. Despite its potential to fulfill these criteria, interoception remains underexplored in MDD. This study aimed to evaluate the potential of interoception in transforming MDD's clinical practices by examining interoception deficits across various MDD stages and analyzing their complex associations with the spectrum of depressive symptoms.

**Methods:**

This study included 431 healthy individuals, 206 subclinical depression individuals, and 483 MDD patients. Depressive symptoms and interoception function were assessed using the PHQ‐9 and MAIA‐2, respectively.

**Results:**

Interoception dysfunction occurred in the preclinical phase of MDD and further impaired in the clinical stage. Antidepressant therapies showed limited efficacy in improving interoception and might damage some dimensions. Interoceptive dimensions might predict depressive symptoms, primarily enhancing negative thinking patterns. The predictive model based on interoception was built with random split verification and demonstrated good discrimination and predictive performance in identifying MDD.

**Conclusions:**

Early alterations in the preclinical stage, multivariate associations with depressive symptoms, and good discrimination and predictive performance highlight the importance of interoception in MDD management, pointing to a paradigm shift in diagnostic and therapeutic approaches.

## Graphical abstract

In this study, interoception impairments, beginning in MDD's preclinical phase and escalating during its clinical stage, were found to predictively impact all primary symptom clusters, particularly emotional symptoms. Internal validation underscored their significant role in improving diagnostic accuracy and predictive capabilities for MDD, which offers discriminative and predictive utility for MDD clinical practice.

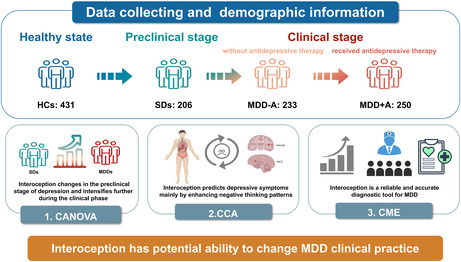

## 1 BACKGROUND

Major depressive disorder (MDD), as a prevalent affective disorder, has experienced a substantial rise in incidence worldwide, becoming a significant global challenge. However, the current diagnostic and treatment protocols for MDD are suboptimal. Primarily, MDD often begins insidiously, presenting initially as vague neurovegetative symptoms such as fatigue, sleeplessness, or unexplained pain,^1^, ^2^ which are frequently interpreted as signs of other medical conditions, causing delays in diagnosis and treatment. Furthermore, the symptom heterogeneity of MDD complicates the accuracy of diagnosis and the understanding its pathophysiological mechanisms. Despite the “Three‐Dimensional Symptom Model” categorizing MDD symptoms into emotional, physical, and cognitive domains as a means to understand MDD's heterogeneity, it primarily emphasizes symptomatic manifestations, neglecting the underlying biological mechanisms.^3^ The subjective nature and clinical overlap of isolated symptoms also limit the efficacy of symptom‐based neurobiological mappings in MDD research.^4^ Finally, the variability in individual responses to antidepressant treatment often leads to persistent residual symptoms like sleep disturbances or fatigue,^5^ adversely affecting patients, quality of life and increasing the risk of recurrence.^6^ Therefore, developing early indicators that integrate emotional, physical, and cognitive dimensions while considering the interplay of physiological, psychological, and environmental factors is crucial for enhancing the diagnosis and treatment outcomes of MDD.

Interoception refers to the perception of the body's internal physiological state, encompassing the interpretation, integration, and regulation of these physiological signals.^7^ Accurate interoceptive perception is essential for homeostatic balance, yet deviations in this process can lead to an exaggerated focus on or misinterpretation of these signals, influencing self‐perception and identity.^8^ Such aberrant interoception may trigger negative emotions like anxiety, tension, and fear, and induce unhealthy physiological responses that lead individuals to adopt maladaptive psychological coping strategies,^9^ making interoception impairment a potential risk factor for psychiatric conditions.^10^

Moreover, the insula, central to the generation and regulation of interoception,^11^ is intricately linked to somatic perception, emotional processing, cognitive functions, and autonomic regulation.^12^, ^13^, ^14^ Therefore, interoception is often explored to explain the emotional, somatic, and cognitive symptoms of various mental and somatic diseases.^15^ Lastly, interoception is influenced by multiple factors, including physiological states, life experiences, and health status, exhibiting strong individualized characteristics.^16^ These features make interoception a promising area to meet the diagnostic and therapeutic needs of MDD. Recent research indicates a potential link between MDD and interoception, with studies showing reduced cardiac interoceptive responses and diminished insular activation during cardiac interoception tasks in MDD patients.^15^, ^16^, ^17^, ^18^, ^19^, ^20^ Previous research has indicated the neural mechanisms underlying interoception and its relationship with peripheral somatic changes in depression include multiple pathways, interactions with individual differences in interoception, and a developmental psychobiological systems perspective.^15^, ^21^ A Meta‐analysis further identified the disrupted dorsal mid‐insula activation as a key neural region in interoception impairment across various psychiatric disorders, including MDD. Notably, mid‐insula cluster differed anatomically from brain regions involved in affective processing and from regions altered by psychological or pharmacological interventions for affective disorders, suggesting interoception as an underexplored potential target for intervention.^22^ Moreover, the interoception neural circuitry, centered around the insula, had been proven to couple with peripheral physiological responses such as systemic inflammatory reactions and glucose metabolism,^23^, ^24^ contributing to depression‐related symptoms like appetite changes,^25^ suicidal and non‐suicidal self‐injurious thoughts and behaviors,^26^ and sleep disturbances.^27^ Researches indicated that improvements in interoception can independently predict positive treatment responses,^28^, ^29^, ^30^ whereas a pre‐treatment decline in bodily signal perception may signal a risk for persistent fatigue symptoms post‐treatment.^30^ The current antidepressants appear to exacerbate abnormalities in bodily sensations within emotional contexts. Enhancing bodily awareness might be an important therapeutic target for MDDs.^31^ These all highlight the value of interoception on MDD.

However, interoception is not limited to cardiac perception but is a multidimensional, multistage psychophysiological process. It shows significant dynamic characteristics, individual differences and sensitivity to antidepressant treatment.^32^ Studies in this area have been limited by small sample sizes and a lack of comprehensive analysis of interoception's multidimensional characteristics across various stages of MDD, hindering the identification of specific clinical questions that interoception may address. They also insufficiently investigated the multivariate relationship between interoception and depressive symptoms, limiting insights into interoception's potential to explain the symptom heterogeneity in MDD. Furthermore, the results of these interoception studies were not internally validated, and there was a distinct lack of evaluation concerning its capacity to discriminate and predict MDD, as well as a clear deficiency in assessing its practical clinical utility. These gaps are necessary steps to validate mental health indicators and their capacity to transform clinical practices.^33^

Therefore, this study aimed to explore the variations in interoception across different stages of MDD, including subclinical and medication‐naïve phases, and how these variations correlate with depressive symptoms. The discrimination and predictive performance of interoception in MDD were also evaluated. Addressing these gaps would clarify the role of interoception in MDD and hold significant theoretical and practical implications for optimizing the diagnosis and treatment of depression. We hypothesized that (1) all dimensions of interoception are impaired in individuals with MDD, evident even in the subclinical phase; (2) interoception primarily contributes to the MDD through affecting somatic symptoms; and (3) measuring multidimensional interoception can help identify MDD, potentially enhancing clinical outcomes.

## 2 MATERIALS AND METHODS

Ethics approval was provided by The Affiliated Mental Health Center of Jiangnan University Ethics Boards (Ethical approval number: WXMHCIRB2021LLky115). All participants provided written informed consent and were compensated 40.00 Chinese Yuan.

### 2.1 Subject recruitment and assessment

A total of 1120 participants (healthy controls [HCs]: 431, subthreshold depression patients [SDs]: 206, MDD patients [MDDs]: 483) were recruited by advertisement in the community and Wuxi Mental Health Center from May 2022 to September 2023.

The inclusions criteria for HC group were (1) age range from 18 to 65 years old; (2) not meeting the criteria for any Diagnostic and Statistical Manual 5th Edition (DSM‐5) axis I disorder or personality disorders, as assessed by the Structured Clinical Interview for DSM‐5; (3) no history of any kind of mental disorder; and (4) no physical illness. The exclusion criteria were (1) pregnant or perinatal women and (2) incomplete assessments.

The inclusions criteria for SD group were (1) age range from 18 to 65 years old; (2) a score of the Patient Health Questionnaire‐9 (PHQ‐9) between 5 and 9^34^; and (3) with a symptom of either depressed mood or loss of interest or pleasure; (4) without any antidepressive therapy. The exclusion criteria were (1) who had suicidal ideation and suicidal attempts; (2) that the duration of symptom were above 2 weeks before enrollment; (3) who received psychotherapy (mindfulness‐based cognitive therapy, etc.) and physical therapy (repetitive transcranial magnetic stimulation, etc.); and (4) returning incomplete assessments.

The inclusions criteria for MDD group were (1) age range from 18 to 65 years old; (2) meeting the MDD criteria for DSM‐5; and (3) that antidepressive therapy duration were no less than 28 days. The exclusion criteria were (1) meeting the criteria for any DSM‐5 axis I disorder or personality disorders, as assessed by the Structured Clinical Interview for DSM‐5; (2) who had a history of any kind of mental disorder; (3) who had any physical illness; (4) who received psychotherapy (mindfulness‐based cognitive therapy, etc.) and physical therapy (repetitive transcranial magnetic stimulation, etc.); and (5) returning incomplete assessments.

**FIGURE 1:**
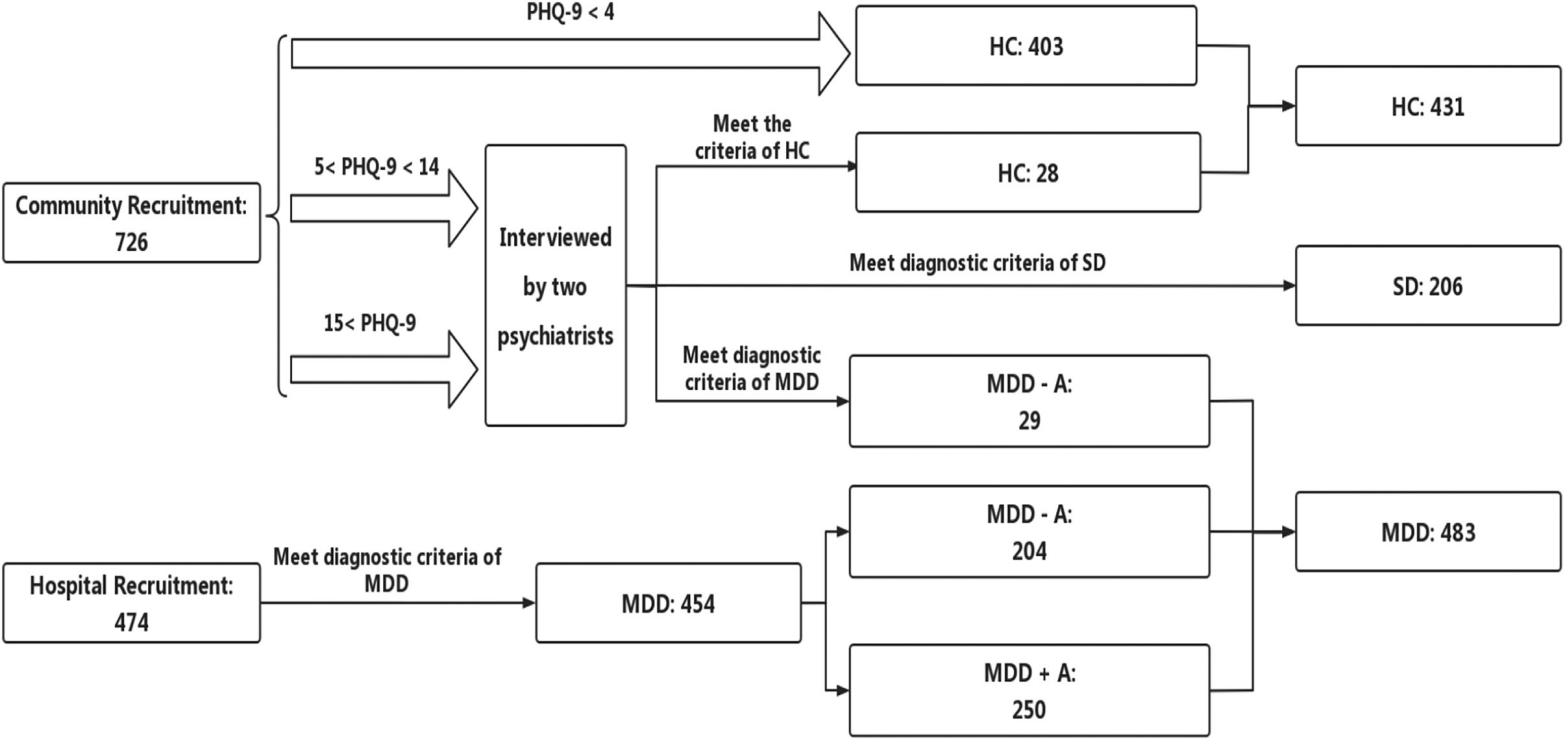
Flow chart. The participants were primarily recruited from community and hospital. Hospital recruitment was overseen by two psychiatrists. Patients diagnosed with major depressive disorder according to the DSM‐5 were included in the study, with their antidepressant treatment status recorded. Community recruitment was mainly conducted by three laboratory staff members, utilizing advertisements. Participants were screened by PHQ‐9. Those with a total PHQ‐9 score exceeding 4 were transferred to hospital outpatient clinics for further evaluation by two psychiatrists. A total of 1120 participants were involved in the study, comprising 431 Healthy controls (HCs), 206 subthreshold depression patients (SDs), 250 MDDs with antidepressive therapy (MDD + A), and 233 MDDs without antidepressive therapy (MDD − A).

All participants were assessed by two psychiatrists. Although there is still no consensus on the definition of SD,^35^ researchers have identified SD as an early stage or precursor of depression.^36^ In this study, SDs were screened using the PHQ‐9 among individuals who self‐identified as HCs. These individuals denied being in a depressive state, perceiving their condition merely as “recoverable stress”. All SDs were re‐examined by a psychiatrist after an initial outpatient assessment. Only those who showed subthreshold depressive symptoms in assessments by two rounds of psychiatrist were included in the SD group (Figure 1).

### 2.2 Measures

The PHQ‐9 and the Generalized Anxiety Disorder‐7 questionnaire (GAD‐7) were used to assess the severity of depression and anxiety.^37^ Multidimensional Assessment of Interoceptive Awareness‐version 2 (MAIA‐2) was applied to assess self‐reported interoception.^38^ The Physical Exercise Rating Scale (PARS‐3) was used to assess the amount of exercise.^39^ Detailed descriptions of these measures are provided in Appendix S1.

### 2.3 Statistical analysis

The statistical analysis consisted of three parts. First, we investigated the changes in interoception at different stages of MDD. The focus was on the pre‐clinical and medication‐naive populations. The groups' differences in demographic information were obtained using the *χ* ^2^ test for categorical variables and the analysis of variance (ANOVA) for continuous variables. Analysis of covariance (ANCOVA) was used to compare interoceptive dimensions across subjects, controlling for a comprehensive set of demographic and lifestyle variables. These covariates included gender, age, body mass index (BMI), years of education, daily exercise duration, time spent on social media, tobacco and alcohol use, coffee and tea consumption, marital status, childbearing status, and total annual household income. Internal consistency reliability for each scale was determined using McDonald's omega or Cronbach's alpha. Post hoc comparisons were corrected using the Bonferroni method. We first compared the differences among three groups. Subsequently, within the depression group, they further subdivided participants into first‐episode, medication‐naïve and previously treated subgroups to analyze the effects of medication. This can effectively reduce the number of comparisons, thereby lowering the Type I error rate (detailed in Appendix S1).

Second, we examined the multivariate relationship between the interoception predictor set and the depression outcome set and determined specific variables within each set that contribute to the strength of this relationship by the Canonical Correlation Analysis (CCA).^40^ The CCA is a statistical method used to explore the relationships between two multivariate sets of variables. It considers multiple variables simultaneously and allows for a more holistic understanding of the relationships between two sets of variables.^41^

**FIGURE 2:**
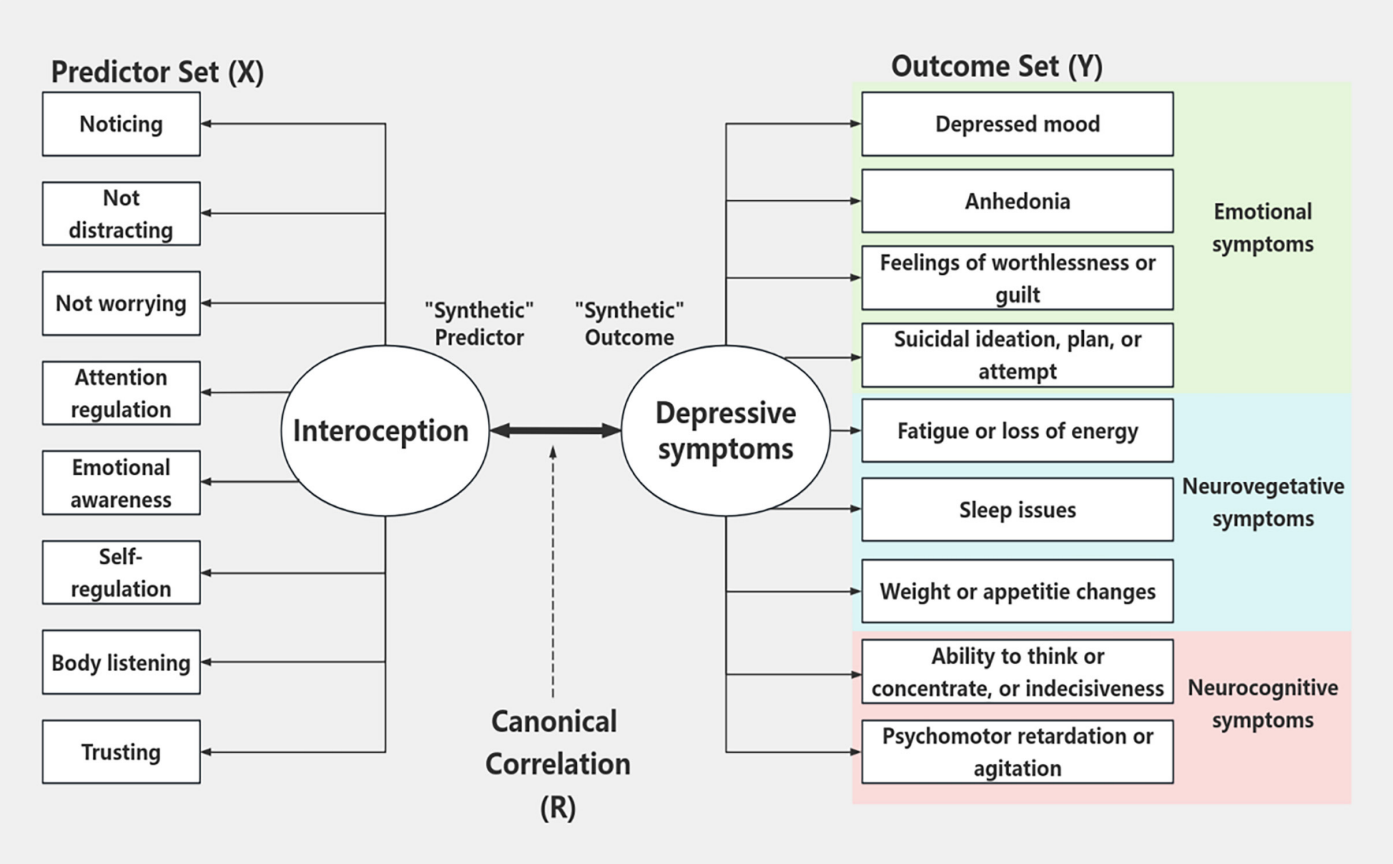
Hypothesized multivariate relationship between the interoception predictors and depressive symptoms outcomes using the Canonical Correlation framework.

In the current study, CCA was applied to evaluate a multivariate relationship between a set of interoception predictors and a set of depression outcomes. Under this framework, we defined the interoception predictor set to consist of eight interoception subscales, and the depression outcomes set to consist of nine depressive symptoms items in PHQ‐9 (Figure 2). Depressive symptoms were divided into emotional symptoms, autonomic symptoms, and neurocognitive symptoms according to previous research.^3^

Finally, we assessed the diagnostic efficacy of interoception for MDD. A clinical prediction model for MDD based on interoception was built using logistic regression analysis. Additionally, a nomogram will be developed based on the logistic regression model. A nomogram is a graphical representation of the logistic regression equation, allowing for the estimation of individual probabilities of depression occurrence based on the values of the predictive factors.^42^, ^43^ The performance of the nomogram was assessed using the receiver operating characteristic (ROC) curve and calibration curve. Decision Curve Analysis (DCA) was also used to evaluate the clinical utility of predictive model (Appendix S2).

The results with a *p*‐value of <0.05 were considered statistically significant. Following the analytical steps recommended by a previous study,^33^ we hoped to provide evidence that interoception had the potential ability to transform MDD's clinical practices. The nomogram was developed using the “rms” package. ROC curves and DCA were constructed using the “pROC” and “riskRegression” packages, respectively. All statistical analyses were performed using the R software (version 4.2.2), along with MSTATA software (www.mstata.com).

## 3 RESULTS

### 3.1 Demographic characteristics

Table 1 presents demographic information. MDDs had fewer years of education (*F* = 91.868, *p* = 0.000) and BMI (*F* = 4.898, *p* = 0.008) than HCs. More MDDs were female and had the habits of smoking and drinking, and spent more time on social media (all *p* < 0.05). Other demographic information had no difference (all *p* > 0.05).

**TABLE 1:** The demographic characteristics of participants.

Characteristics	Participants			
	HC (*n* = 431^a^)	SD (*n* = 206^a^)	MDD (*n* = 483^a^)	*p*‐Value^b^
Age, mean (SD), years	28 (9)	26 (8)	27 (10)	0.207
Gender, female (%)	262 (61%)	132 (64%)	345 (71%)	0.003
Education, mean (SD), years	15.8 (3.1)	15.6 (2.9)	13.3 (3.0)	<0.001
Body mass index, mean (SD)	22.5 (3.3)	21.7 (2.9)	21.9 (3.3)	0.008
Physical exercise, mean (SD), h/day	12 (14)	11 (13)	10 (15)	0.268
Marital status (%)				
Married	122 (28%)	48 (23%)	146 (30%)	0.140
Single	301 (70%)	153 (74%)	319 (66%)	
Divorced	8 (2%)	5 (2%)	18 (4%)	
Pregnancy, number (%)				
0	388 (90%)	178 (86%)	377 (78%)	<0.001
1	29 (7%)	20 (10%)	85 (18%)	
2	13 (3%)	6 (3%)	19 (4%)	
3	1 (0)	2 (1%)	2 (0)	
Frequency for cigarette smoking in the past month (%)				
No smoking	388 (90%)	185 (90%)	400 (83%)	0.007
>7 days	6 (1%)	8 (4%)	16 (3%)	
2–7 days	19 (4%)	4 (2%)	34 (7%)	
Everyday	18 (4%)	9 (4%)	33 (7%)	
Frequency for coffee and tea intake in the past month (%)				
No coffee and tea intake	263 (61%)	131 (64%)	317 (66%)	0.310
>7 days	96 (22%)	50 (24%)	103 (21%)	
2–7 days	43 (10%)	19 (9%)	44 (9%)	
Everyday	29 (7%)	6 (3%)	19 (4%)	
Frequency for alcohol drinkers in the past month (%)				
No drinking	382 (89%)	182 (88%)	414 (86%)	0.013
>7 days	39 (9%)	24 (12%)	51 (11%)	
2–7 days	7 (2%)	0 (0%)	4 (1%)	
Everyday	3 (1%)	0 (0%)	14 (3%)	
Daily social media usage time, hours (%)				
<3	80 (19%)	28 (14%)	86 (18%)	0.004
3–5	160 (37%)	87 (42%)	154 (32%)	
5–8	117 (27%)	53 (26%)	113 (23%)	
>8	74 (17%)	38 (18%)	130 (27%)	
Economic status (annual family income), Chinese Yuan (%)				
<10,000	191 (44%)	84 (41%)	122 (25%)	<0.001
10,000–30,000	205 (48%)	108 (52%)	299 (62%)	
30,000–50,000	26 (6%)	8 (4%)	45 (9%)	
>50,000	9 (2%)	6 (3%)	17 (4%)	
Noticing, mean (SD)	2.35 (1.18)	2.58 (1.05)	2.74 (1.03)	<0.001
Not distracting, mean (SD)	3.09 (1.10)	2.84 (0.97)	2.83 (1.04)	<0.001
Not worrying, mean (SD)	2.65 (0.75)	2.40 (0.72)	2.05 (0.93)	<0.001
Attention regulation, mean (SD)	2.23 (1.10)	2.30 (0.85)	2.00 (0.93)	<0.001
Emotional awareness, mean (SD)	2.35 (1.29)	2.52 (1.01)	2.44 (1.12)	0.224
Self‐regulation, mean (SD)	2.24 (1.22)	2.02 (1.02)	1.31 (0.96)	<0.001
Body listening, mean (SD)	1.99 (1.29)	1.97 (1.00)	1.54 (1.12)	<0.001
Trusting, mean (SD)	2.52 (1.30)	2.29 (1.00)	1.65 (0.98)	<0.001
PHQ9, mean (SD)	1 (1)	7 (1)	19 (5)	<0.001
GAD7, mean (SD)	1.0 (1.9)	6.2 (3.6)	13.1 (4.8)	<0.001
Disease duration, mean (SD), months	—	—	31 (45)	—

Abbreviation: SD, standard deviation.

^a^ *n* (%).

^b^ One‐way ANOVA; Pearson's Chi‐squared test; Fisher's exact test.

### 3.2 Interoception in MDD

#### 3.2.1 Interoception changes in different stages of MDD

**FIGURE 3:**
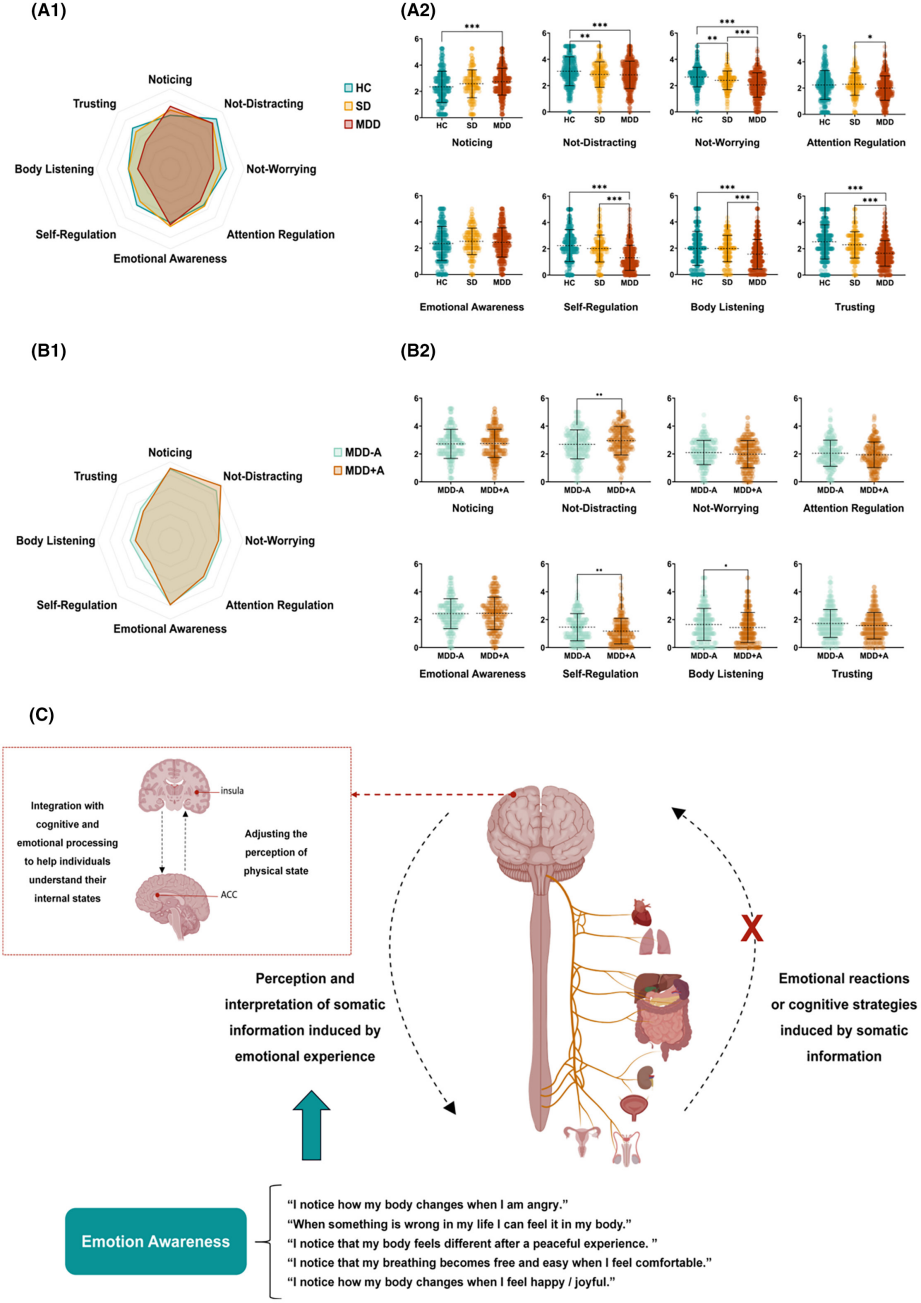
Interoception changes in different stages of MDD. Picture A demonstrated the multidimensional interoceptive features across three groups: HCs, SDs, and MDDs. Picture A1 demonstrated a notable trend in the variation of interoception dimensions when comparing SD and MDD to HC. This trend was characterized by an escalation in “Noticing” and a modest increase in “Emotional Awareness”, whereas other interoception dimensions exhibited a decline. Notably, this pattern was more pronounced in MDDs compared with SDs, highlighting the profound impact of MDD progression on an individual's interoception capability. Picture A2 presented scatter plots for HC, SD, and MDD groups. The mean and standard deviation for the eight interoception dimensions are shown in Table 1. After controlling for demographic variables, significant differences were observed in all interoception dimensions except emotional awareness across the three groups, indicating MDDs had no difference in the somatic perception and interpretation induced by emotion. Picture B revealed the multidimensional interoception characteristics of MDDs with antidepressive therapy (MDD + A) versus MDDs without antidepressive therapy (MDD − A). Picture B1 illustrates that there are no differences in noticing and emotional awareness between MDD + A and MDD − A. Except for improving Not‐Distracting, antidepressants tended to decrease other interoception dimensions. Picture B2 was a scatter plot for MDD − A and MDD + A. The mean and standard deviation for the eight interoception dimensions are shown in Appendix S2. Although there was a notable improvement in the “Not‐Distracting” post‐treatment (mean [2.69–2.95], SD [1.04–1.03]), we observed a decreased effect on “Self‐Regulation” (mean [1.46–1.18], SD [0.98–0.92]) and “Body Listening” (mean [1.66–1.44], SD [1.15–1.08]). This highlighted the need for a more individualized approach in the treatment of MDD, where the specific deficits in interoception were considered. Picture C illustrated the transmission process of interoception signals within the human body. The sensory signals from the heart, respiratory system, and other visceral organs were initially conveyed to the insular cortex via the vagus nerve. In the insular cortex, these signals underwent preliminary neural processing, transforming into more complex neural signals. These processed interoceptive signals were subsequently relayed to the anterior cingulate cortex (ACC), where they are integrated with emotional, somatic, and cognitive information. The integrated signals were then transmitted from the insular cortex to the peripheral system, regulating and controlling peripheral physiological responses. “Emotional Awareness” referred to an individual's perception and understanding of bodily signal changes under various emotional states while other interoception dimensions reflected the processing and responding of brain to bodily states changes. “Emotion Awareness” was no significant difference in groups, which suggested that the perception and interpretation of somatic information induced by the emotional experience were similar among groups. Interoception encompasses the cognitive and physiological processes involved in perceiving, interpreting, integrating, and regulating internal bodily signals.^80^ This result was particularly noted because it indicated that the interoception impairments in MDD might not in the signaling output phase. ***p < 0.001, **p < 0.01, *p < 0.05.

Adjusting for all demographic variables, our analysis revealed a trended alteration in interoception from HCs to SDs, and further to MDDs. Although SDs demonstrated increased “noticing” alongside decreased “self‐regulation” and “trusting” compared with HCs, these differences did not reach statistical significance. In contrast, among MDDs, notable interoception disparities emerged, with “noticing” (*F* = 11.78, *p* < 0.001, *η* ^2^ = 0.021), “self‐regulation” (*F* = 59.68, *p* < 0.001, *η* ^2^ = 0.097), and “trusting” (*F* = 44.35, *p* < 0.001, *η* ^2^ = 0.074) significantly diverging from HCs. Moreover, MDDs exhibited significant reductions in “attention regulation” (*F* = 4.68, *p* = 0.009, *η* ^2^ = 0.008) and “body listening” (*F* = 12.032, *p* < 0.001, *η* ^2^ = 0.021) compared with both SDs and HCs. SDs and MDDs, who did not differ significantly from each other, performed markedly worse in “not distracting” than HCs (*F* = 10.56, *p* < 0.001, *η* ^2^ = 0.019). A gradual decline in “not worrying” was observed from HCs to SDs to MDDs (*F* = 44.28, *p* < 0.001, *η* ^2^ = 0.074). However, we did found the difference in “emotional awareness” (*F* = 2.00, *p* = 0.136, *η* ^2^ = 0.004) (Figure 3A,C).

#### 3.2.2 Effects of antidepressive therapy on interoception in MDDs

We further grouped the MDDs by with/without antidepressive therapy. There were 250 MDDs with antidepressive therapy (MDD + A) and 233 MDDs without antidepressive therapy (MDD − A). For MDDs, the antidepressive therapy duration was 36 days (Standard deviation [SD] [6]). According to the previous studies,^44^, ^45^ the mean Fluoxetine‐equivalent dose of antidepressants was 37.2 (SD [1.2]) mg/day, and the mean chlorpromazine‐equivalent dose of antipsychotics was 348.6 (SD [95.1]) mg/day. The demographics and baseline characteristics of MDD − A and MDD + A, and antidepressive agents for MDD + A are listed in Appendix S3.

After controlling for all demographic characteristics, disease duration, and anxiety, MDD + A showed improved “not distracting” (*F* = 9.36, *p* = 0.002, *η* ^*2*^ = 0.020), and further exacerbated “self‐regulation” (*F* = 8.57, *p* = 0.004, *η* ^*2*^ < 0.001) and “body listening” (*F* = 6.03, *p* = 0.014, *η* ^*2*^ = 0.013) than MDD − A. Other dimensions had no significant differences between two groups (all *p* > 0.05) (Figure 3B).

#### 3.2.3 Interoception predict depressive symptoms

The CCA was conducted to evaluate the multivariate shared relationship between the interoception predictors set and depressive symptoms outcomes set. The correlation coefficients of the first pair of canonical variates were statistically significant (*p* < 0.05), with the first correlation coefficient having a value of 0.394, an eigenvalue of 0.184, a contribution rate of 60.53% (*F* = 1.91, *p* < 0.001). The effect size (1 − Λ) for the full model was 0.155, suggesting that a substantial 15.5% of variance was shared between interoception dimensions and depressive symptoms.

Subsequently, the structure coefficients (*r*_s_) and squared structure coefficients ($r_{s}^{2}$) of individual variables were evaluated to determine which variables contributed to the relationships between sets of variables. $r_{s}^{2}$ of emotional symptoms, neurovegetative symptoms, and neurocognitive symptoms were 137.09, 87.98, and 56.45.^3^

**FIGURE 4:**
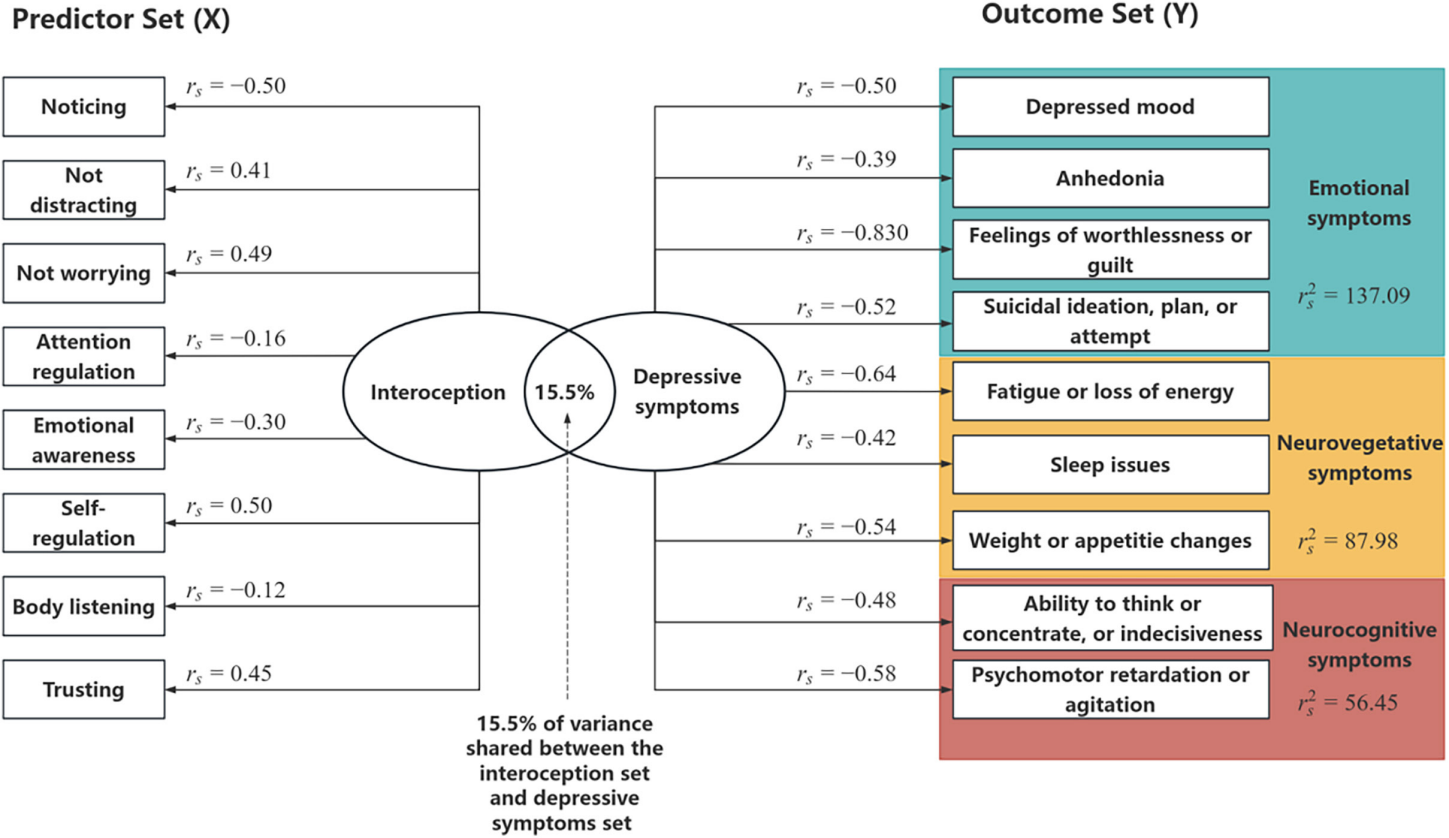
Multivariate relationship between the interoception predictors and depressive outcomes. The *r*_s_ values represent structure coefficients.

“Self‐regulation”, “noticing” and “not worrying” were the primary drivers among interoception dimensions, whereas PHQ6 from emotional symptoms ($r_{s}^{2}$ = 69.22), PHQ4 from neurovegetative symptoms ($r_{s}^{2}$ = 41.47), and PHQ8 from neurocognitive symptoms ($r_{s}^{2}$ = 33.41) were the primary drivers among the depressive symptoms (Table 2 and Figure 4).

**TABLE 2:** Canonical solution for interoception dimensions set (predictors in the model) and the depressive symptoms outcomes set (outcomes in the model).

Variables	Full model			Symptoms ($r_{s}^{2}$)
	Coef	*r*_s_	$r_{s}^{2}$ (%)	
Outcomes (depressive symptoms)				
PHQ1: Depressed mood	−0.10	−0.50	25.00	Emotional symptoms (137.09)
PHQ2: Anhedonia	0.24	−0.39	15.52	
PHQ6: Feelings of worthlessness or guilt	−0.60	−0.83	69.22	
PHQ9: Suicidal ideation, plan, or attempt	−0.09	−0.52	27.35	
PHQ3: Sleep issue	−0.12	−0.42	17.89	Neurovegetative symptoms (87.98)
PHQ4: Fatigue or loss of energy	−0.30	−0.64	41.47	
PHQ5: Weight or appetite changes	−0.20	−0.54	28.62	
PHQ7: Ability to think or concentrate, or indecisiveness	−0.05	−0.48	23.04	Neurocognitive symptoms (56.45)
PHQ8: Psychomotor retardation or agitation	−0.23	−0.58	33.41	
Predictors (interoception dimensions)				
Noticing	−0.26	−0.50	24.50	
Not distracting	0.35	0.41	17.06	
Not worrying	0.19	0.49	24.11	
Attention regulation	−0.23	−0.16	2.56	
Emotional awareness	−0.22	−0.30	8.76	
Self‐regulation	0.67	0.50	25.10	
Body listening	−0.19	−0.12	1.49	
Trusting	0.40	0.45	19.98	

*Note*: The categorization of the depressive symptoms include Emotional symptoms, Neurovegetative symptoms, and Neurocognitive symptoms. Coef, standardized canonical function coefficient; *r*_s_, structure coefficient; $r_{s}^{2}$, squared structure coefficient—percent of variance shared between the individual variable and the variable set.

#### 3.2.4 Diagnostic efficacy of interoception for MDD

**FIGURE 5:**
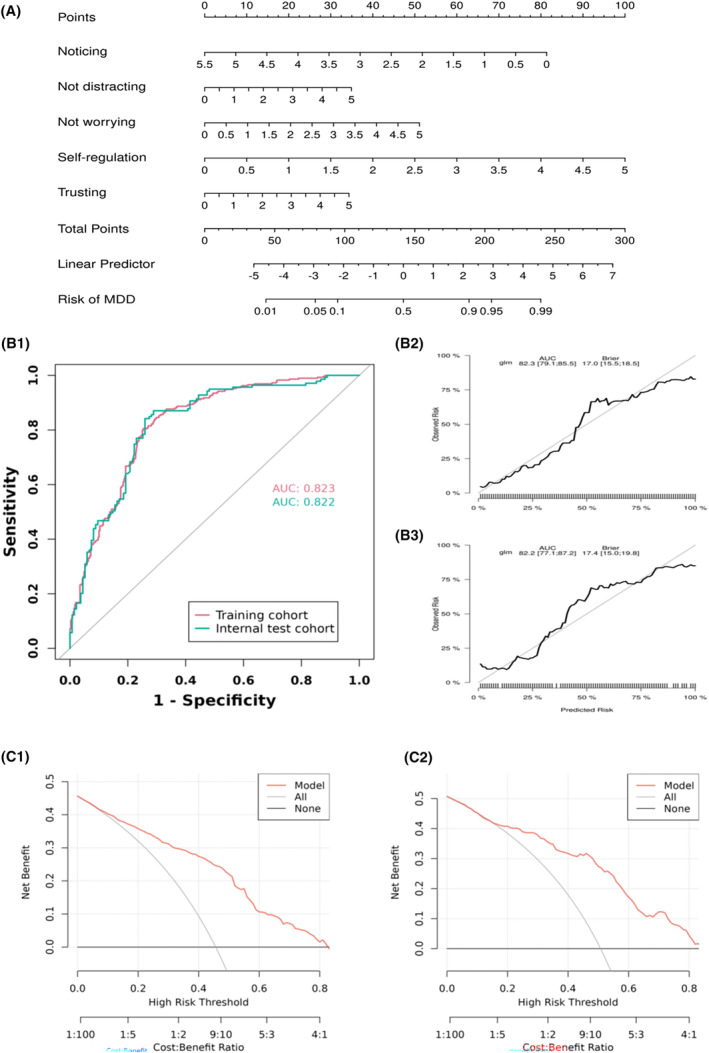
Diagnostic efficacy of interoception for MDD. Picture A: Nomogram prediction model. Picture B: ROC curves of the nomogram prediction model. The prediction model showed a good classification ability. Picture B: the ROC curves of the nomogram prediction model (B1). The prediction model showed a good classification ability. calibration curve of the nomogram prediction mode for the training cohort (B2) and the internal test cohort (B3). The results showed that the original nomogram was still valid for use in the validation sets and the calibration curve of this model was relatively close to the ideal curve, which indicates that the predicted results were consistent with the actual findings. Picture C: DCA curve of the nomogram of the training cohort (C1) and the internal test cohort (C2). A high‐risk threshold probability indicated the chance of significant discrepancies in the model's prediction when clinicians encountered major flaws while utilizing the nomogram for diagnostic and decision‐making purposes. This research showed that the nomogram offered substantial net benefits for clinical application through its DCA curve.

Five potential predictors (noticing, not distracting, not worrying, self‐regulation, body listening, and trusting) were selected by LASSO regression analysis (Appendix S2). The final logistic model is developed as a simple‐to‐use nomogram, which is illustrated in Figure 5A. ROC analysis revealed that AUC value of the nomogram reached 0.823 (CI: 0.795–0.858) in training cohort and 0.822 (CI: 0.796–0.890) in internal test cohort, indicating that this model has stable and good discriminant ability (Figure 5B1). The calibration plots of the nomogram in the different cohorts demonstrated a good correlation between the observed and predicted MDD status (Figure 5B2,B3). DCA curves indicated that using this model is better than no intervention at all for all threshold levels in both cohorts, showing that the nomogram is effective in clinical practice (Figure 5C).

## 4 DISCUSSION

This is the largest study to date investigating interoception deficits at different stages of MDD and their correlation with depressive symptoms. Previous research on interoception in MDD was constrained, particularly in understanding interoception dysfunction during various MDD stages. Furthermore, the multidimensional nature and individual variability of interoception also complicated the research.^46^, ^47^, ^48^ Our study addresses these gaps by focusing subclinical and drug‐naïve MDD population and collecting more comprehensive demographic data. We found that individuals demonstrated interoceptive deficits prior to realizing their depressive state (in SD stage), characterized by heightened concern for unpleasant sensations and a reduced capacity to distract attention from discomfort. In the clinical stage of MDD, the range of interoception impairment dimensions was more severe. Specifically, MDDs were hypersensitive to body sensations (higher “noticing”), unable to focus on and regulate physical sensations (lower “attention regulation” and “self‐regulation”) and had difficulty in interpreting and trusting bodily cues (“lower body listening” and “trusting”). The interoception dysfunction in various stages made it an early diagnostic and intervention marker for MDD. Additionally, it could also explain the observed efficacy of mindfulness‐based cognitive therapy (MBCT), which is rooted in interoceptive principles, in treating SD and MDD, thereby substantiating the role of interoception in MDD management.^49^, ^50^, ^51^, ^52^ However, the interoception dysfunction identified in MDDs partially diverged from prior research. While reductions in “body listening” and “trusting” were consistent with earlier study, we observed an increased notice to bodily signals in MDDs, which contrary to the findings of Dunne et al.^53^ Existing literature highlighted that MDDs exhibited heightened pain sensitivity,^54^, ^55^ and were prone to comorbid interoception dysfunction conditions like irritable bowel syndrome.^56^, ^57^ Animal experiments also showed the enhanced pain sensitivity in mice under long‐term chronic stress.^58^, ^59^ Consequently, we concluded that MDDs should have a heightened capacity for bodily information perception. It is noteworthy that Dunne's research focused on depressive symptoms in primary care patients, limiting its applicability across different populations and healthcare setting.^53^ Interestingly, our study revealed that while MDDs exhibited slight higher levels of “emotion awareness” compared with HCs, the difference was not statistically significant and appeared unrelated to medication. This indicated that MDDs had stronger emotional reactions to physical discomfort (significance in “no worrying”), but their perception and interpretation of somatic information induced by the purely emotional experience might be similar to HCs (no significance in “emotion awareness”). Bodily experiences could play a more significant role in contributing to MDDs' emotional problems than we previously thought.^60^ Residual somatic symptoms have always been a difficulty in antidepressant treatment and an important factor in the recurrence of depression.^52^, ^53^, ^61^, ^62^ Our findings suggest that strategies relying solely on emotional therapy to alleviate somatic symptoms might have limited efficacy. Further research is required to comprehensively understand the connection between mind and body.^62^

In the investigation of antidepressant treatment effects on interoception, results showed that MDD with antidepressive therapy were more likely to disregard uncomfortable bodily sensations compared with without antidepressive therapy. However, their ability to listen to the body for cues or insight and calming one's own down by paying attention to bodily information were further impaired. These results suggested that MDDs with antidepressive therapy exhibited a blunted interoceptive pattern. While this blunted interoception might alleviate excessive focus on uncomfortable bodily sensations, it seemed to exacerbate the impairment of other interoceptive regulation capabilities. Lyons' research yielded similar conclusions,^31^ indicating that treated MDDs have weaker somatic responses in emotional contexts. Antidepressants, particularly selective serotonin reuptake inhibitors (SSRIs), are known to inhibit exteroceptive processing,^63^, ^64^ alleviate visceral pain,^65^ increase emotional “blunting”,^66^ reduce responses to reward and punishment,^67^ and increase amygdala and insular responses to threat cues.^68^ These evidences highlight the inhibitory effect of antidepressants on the general neural response to interoception.^69^ Additionally, it suggests that current antidepressive therapy may achieve their effects by damaging interoceptive functions. Therefore, investigating the interoceptive functions in MDD is essential. Integrating new interoceptive‐based therapeutic interventions could be a potentially effective strategy for MDD treatment and prevention.

Currently, intervention strategies targeting interoception have shown promising results. MBCT effectively enhances emotional regulation and psychological resilience by increasing interoceptive awareness.^70^, ^71^ However, its efficacy may be limited in MDDs during acute episodes due to impaired meditation ability.^72^, ^73^ Conversely, body psychotherapy and massage therapy, which enhance the mind–body connection through physical interventions,^74^, ^75^, ^76^, ^77^ have demonstrated significant efficacy in managing MDD. It indicates that bodily engagement in the treatment can directly influence mental health. Based on this, Lyons et al.^60^ introduced an innovative interview technique aimed at exploring bodily experiences related to MDD. This technique focuses on promoting deep introspection of somatic sensations in MDDs, providing further insights into how bodily experiences shape depressive episodes. These findings underscored the clinical utility of interoception research in depression.

We further identified a multivariate correlation between interoception and depressive symptoms. Contrary to our hypothesis that interoception impacted depression through somatic symptoms, interoceptive dimensions were found to affect the breadth of depressive symptoms, illustrating the potential of interoception in explaining MDD's symptomatic heterogeneity. In the current research, interoception primarily accounted for emotional symptoms, particularly feelings of worthlessness or guilt.^17^, ^78^ This indicated that interoception mainly lead to MDD by enhancing negative thinking patterns,^47^ aligning with our prior neurophysiological study's findings, i.e., the altered cardiac interoception contributed to the aberrant encoding of negative emotional faces in MDD, mediated by atypical activation in the right insula and anterior cingulate cortex (ACC).^79^ This clinical study reinforced these experimental results that indicated interoception is involved in the pathogenesis of MDD.

Ultimately, our study evaluated the role of interoception in identifying MDD with an internal validation. Key interoceptive factors—self‐regulation, noticing, not worrying, trust, and not distracting—reliably separated MDDs from HCs in our datasets. These dimensions were also identified as the major five interoception predictors of depression within CCA. Additionally, we used DCA to supplementarily assess the utility of the model in actual clinical decision‐making. The results reaffirmed the role of interoception in improving the clinical net benefit for MDDs.

In summary, we effectively tested the ability of interoception to change clinical practice in MDD according to a standardized process.^34^ Our findings highlighted the importance of considering interoception as integral to the diagnosis and treatment of MDD. Future research on interoception's brain mechanisms in MDD and the creation of targeted clinical interventions are vital for better diagnosis and management of MDD. Importantly, from the perspective of neuroimaging and neuroelectrophysiology, studies are needed to further clarify the role of interoception in the pathogenesis of MDD.

The study had three principal limitations. First, its observational nature limited understanding of interoception's role in MDD, indicating the need for future large‐scale, longitudinal, and multi‐center studies by a prospective cohort design. Second, the present research did not extend to interventional studies on interoception in depression. Supplementing with intervention research will help us further clarify the role of interoception in MDD. Given the potential impairment of interoception, future research should focus on the effects of different antidepressants on interoception. Finally, the impact of demographic differences, such as gender and cultural variations, requires detailed investigation in the future. Examining specific bodily experiences (e.g., head, stomach, heart) could also provide deeper insights into the role of bodily information in the pathophysiology of depression.

## 5 CONCLUSIONS

Our findings demonstrated a potential progressive exacerbation of interoception impairments throughout MDD progression, suggesting their potential as early markers for diagnosis and intervention. Given the modest impact of traditional antidepressive therapies on these impairments, our findings highlighted the need for integrating interoception into treatment strategies. Our study also identified a complex multivariate correlation between interoception and depressive symptoms, advancing our understanding of the complex physiological–psychological interplay in MDD pathogenesis which shed light on depression's heterogeneity. Importantly, internal validation confirmed the strong discrimination and predictive power of interoception, reinforcing its value in refining diagnostic accuracy and personalizing treatment. Overall, our findings highlighted the importance of interoception in MDD management, pointing towards a paradigm shift in diagnostic and therapeutic approaches.

## AUTHOR CONTRIBUTIONS

We extend our sincere gratitude to Zhenhe Zhou for his financial support. Hongliang Zhou played a pivotal role in conceptualizing the study, analyzing data, and drafting the manuscript. Chenguang Jiang contributed to the manuscript writing. Jikang Liu and Yuqing Wu were responsible for recruiting depression subjects and conducting psychiatric assessments. Zixuan Huang, Wenliang Wang, Yuhang Ma undertook the community recruitment.

## FUNDING INFORMATION

This study was supported in part by grant no. 202107 from Wuxi Municipal Health Commission Major Project. The funder approved the study protocol prior to study initiation. The funder had no role in the conduct of the study; collection, management, analysis, and interpretation of the data; preparation, review, or approval of the manuscript; and decision to submit the manuscript for publication.

## CONFLICT OF INTEREST STATEMENT

The authors declare no conflicts of interest.

## Supporting information


Appendix S1

Appendix S2

Appendix S3

## Data Availability

Data will be available to researchers with a signed data access agreement. Data will be made available to researchers whose proposed use of the data has been approved. Data will be made available for any purpose. Data will be available to researchers by hongliangzh2022@hotmail.com.

## References

[1] Liao Y , Zhang H , Guo L , et al. Impact of cognitive‐affective and somatic symptoms in subthreshold depression transition in adults: evidence from depression cohort in China (DCC). J Affect Disord. 2022;315:274‐281. 35952931 10.1016/j.jad.2022.08.009

[2] Crouse JJ , Carpenter JS , Song YJC , et al. Circadian rhythm sleep‐wake disturbances and depression in young people: implications for prevention and early intervention. Lancet Psychiatry. 2021;8(9):813‐823. 34419186 10.1016/S2215-0366(21)00034-1

[3] Malhi GS , Mann JJ . Depression. Lancet. 2018;392(10161):2299‐2312. 30396512 10.1016/S0140-6736(18)31948-2

[4] Kroemer NB , Opel N , Teckentrup V , et al. Functional connectivity of the nucleus accumbens and changes in appetite in patients with depression. JAMA Psychiatry. 2022;79(10):993‐1003. 36001327 10.1001/jamapsychiatry.2022.2464 PMC9403857

[5] Xiao L , Feng L , Zhu XQ , et al. Comparison of residual depressive symptoms and functional impairment between fully and partially remitted patients with major depressive disorder: a multicenter study. Psychiatry Res. 2018;261:547‐553. 29407721 10.1016/j.psychres.2018.01.020

[6] Buckman JEJ , Underwood A , Clarke K , et al. Risk factors for relapse and recurrence of depression in adults and how they operate: a four‐phase systematic review and meta‐synthesis. Clin Psychol Rev. 2018;64:13‐38. 30075313 10.1016/j.cpr.2018.07.005 PMC6237833

[7] Chen WG , Schloesser D , Arensdorf AM , et al. The emerging science of interoception: sensing, integrating, interpreting, and regulating signals within the self. Trends Neurosci. 2021;44(1):3‐16. 33378655 10.1016/j.tins.2020.10.007 PMC7780231

[8] Park HD , Blanke O . Coupling inner and outer body for self‐consciousness. Trends Cogn Sci. 2019;23(5):377‐388. 30826212 10.1016/j.tics.2019.02.002

[9] Petzschner FH , Garfinkel SN , Paulus MP , Koch C , Khalsa SS . Computational models of interoception and body regulation. Trends Neurosci. 2021;44(1):63‐76. 33378658 10.1016/j.tins.2020.09.012 PMC8109616

[10] Brewer R , Murphy J , Bird G . Atypical interoception as a common risk factor for psychopathology: a review. Neurosci Biobehav Rev. 2021;130:470‐508. 34358578 10.1016/j.neubiorev.2021.07.036 PMC8522807

[11] Berntson GG , Khalsa SS . Neural circuits of interoception. Trends Neurosci. 2021;44(1):17‐28. 33378653 10.1016/j.tins.2020.09.011 PMC8054704

[12] Livneh Y , Sugden AU , Madara JC , et al. Estimation of current and future physiological states in insular cortex. Neuron. 2020;105(6):1094‐1111.e10. 31955944 10.1016/j.neuron.2019.12.027 PMC7083695

[13] Tallon‐Baudry C . Interoception: probing internal state is inherent to perception and cognition. Neuron. 2023;111(12):1854‐1857. 37160115 10.1016/j.neuron.2023.04.019

[14] Gu X , FitzGerald TH . Interoceptive inference: homeostasis and decision‐making. Trends Cogn Sci. 2014;18(6):269‐270. 24582825 10.1016/j.tics.2014.02.001

[15] Harshaw C . Interoceptive dysfunction: toward an integrated framework for understanding somatic and affective disturbance in depression. Psychol Bull. 2015;141(2):311‐363. 25365763 10.1037/a0038101 PMC4346391

[16] Bonaz B , Lane RD , Oshinsky ML , et al. Diseases, disorders, and comorbidities of interoception. Trends Neurosci. 2021;44(1):39‐51. 33378656 10.1016/j.tins.2020.09.009

[17] Khalsa SS , Adolphs R , Cameron OG , et al. Interoception and mental health: a roadmap. Biol Psychiatry Cogn Neurosci Neuroimaging. 2018;3(6):501‐513. 29884281 10.1016/j.bpsc.2017.12.004 PMC6054486

[18] Terhaar J , Viola FC , Bär KJ , Debener S . Heartbeat evoked potentials mirror altered body perception in depressed patients. Clin Neurophysiol. 2012;123(10):1950‐1957. 22541740 10.1016/j.clinph.2012.02.086

[19] Ishii R , Canuet L . Heartbeat evoked potentials: a new possible clinical biomarker for depression based on the somatic marker hypothesis. Clin Neurophysiol. 2012;123(10):1899‐1900. 22502951 10.1016/j.clinph.2012.03.002

[20] Wiebking C , de Greck M , Duncan NW , Tempelmann C , Bajbouj M , Northoff G . Interoception in insula subregions as a possible state marker for depression – an exploratory fMRI study investigating healthy, depressed and remitted participants. Front Behav Neurosci. 2015;9:82. 25914633 10.3389/fnbeh.2015.00082 PMC4392695

[21] Hu L , He H , Roberts N , et al. Insular dysfunction of interoception in major depressive disorder: from the perspective of neuroimaging. Front Psych. 2023;14:1273439. 10.3389/fpsyt.2023.1273439 PMC10568471 37840807

[22] Nord CL , Lawson RP , Dalgleish T . Disrupted dorsal mid‐insula activation during interoception across psychiatric disorders. Am J Psychiatry. 2021;178(8):761‐770. 34154372 10.1176/appi.ajp.2020.20091340 PMC7613124

[23] Aruldass AR , Kitzbichler MG , Morgan SE , et al. Dysconnectivity of a brain functional network was associated with blood inflammatory markers in depression. Brain Behav Immun. 2021;98:299‐309. 34450247 10.1016/j.bbi.2021.08.226

[24] Hannestad J , Subramanyam K , Dellagioia N , et al. Glucose metabolism in the insula and cingulate is affected by systemic inflammation in humans. J Nucl Med. 2012;53(4):601‐607. 22414635 10.2967/jnumed.111.097014 PMC3717572

[25] Cosgrove KT , Burrows K , Avery JA , et al. Appetite change profiles in depression exhibit differential relationships between systemic inflammation and activity in reward and interoceptive neurocircuitry. Brain Behav Immun. 2020;83:163‐171. 31604141 10.1016/j.bbi.2019.10.006 PMC6937709

[26] Ho TC , Walker JC , Teresi GI , et al. Default mode and salience network alterations in suicidal and non‐suicidal self‐injurious thoughts and behaviors in adolescents with depression. Transl Psychiatry. 2021;11(1):38. 33436537 10.1038/s41398-020-01103-x PMC7804956

[27] Li G , Chen Y , Chaudhary S , et al. Sleep dysfunction mediates the relationship between hypothalamic‐insula connectivity and anxiety‐depression symptom severity bidirectionally in young adults. Neuroimage. 2023;279:120340. 37611815 10.1016/j.neuroimage.2023.120340

[28] Billones RR , Kumar S , Saligan LN . Disentangling fatigue from anhedonia: a scoping review. Transl Psychiatry. 2020;10:273. 32769967 10.1038/s41398-020-00960-w PMC7414881

[29] Zwienenberg L , van Dijk H , Enriquez‐Geppert S , et al. Heartbeat‐evoked potential in major depressive disorder: a biomarker for differential treatment prediction between venlafaxine and rTMS? Neuropsychobiology. 2023;82(3):158‐167. 36927872 10.1159/000529308

[30] Eggart M , Valdés‐Stauber J , Müller‐Oerlinghausen B , Heinze M . Dysfunctional self‐reported interoception predicts residual symptom burden of fatigue in major depressive disorder: an observational study. BMC Psychiatry. 2023;23(1):667. 37700276 10.1186/s12888-023-05168-y PMC10498532

[31] Lyons N , Strasser A , Beitz B , et al. Bodily maps of emotion in major depressive disorder. Cogn Ther Res. 2021;45:508‐516.

[32] Nord CL , Garfinkel SN . Interoceptive pathways to understand and treat mental health conditions. Trends Cogn Sci. 2022;26(6):499‐513. 35466044 10.1016/j.tics.2022.03.004

[33] Abi‐Dargham A , Moeller SJ , Ali F , et al. Candidate biomarkers in psychiatric disorders: state of the field. World Psychiatry. 2023;22(2):236‐262. 37159365 10.1002/wps.21078 PMC10168176

[34] Richardson LP , McCauley E , Grossman DC , et al. Evaluation of the Patient Health Questionnaire‐9 item for detecting major depression among adolescents. Pediatrics. 2010;126(6):1117‐1123. 21041282 10.1542/peds.2010-0852 PMC3217785

[35] Noyes BK , Munoz DP , Khalid‐Khan S , Brietzke E , Booij L . Is subthreshold depression in adolescence clinically relevant? J Affect Disord. 2022;309:123‐1230. 35429521 10.1016/j.jad.2022.04.067

[36] Zhang B , Liu S , Liu X , et al. Discriminating subclinical depression from major depression using multi‐scale brain functional features: a radiomics analysis. J Affect Disord. 2022;297:542‐552. 34744016 10.1016/j.jad.2021.10.122

[37] Karyotaki E , Efthimiou O , Miguel C , et al. Internet‐based cognitive behavioral therapy for depression: a systematic review and individual patient data network meta‐analysis. JAMA Psychiatry. 2021;78(4):361‐371. 33471111 10.1001/jamapsychiatry.2020.4364 PMC8027916

[38] Teng B , Wang D , Su C , et al. The multidimensional assessment of interoceptive awareness, version 2: translation and psychometric properties of the Chinese version. Front Psych. 2022;13:970982. 10.3389/fpsyt.2022.970982 PMC9691670 36440402

[39] Fu W , Li Y , Liu Y , et al. The influence of different physical exercise amounts on learning burnout in adolescents: the mediating effect of self‐efficacy. Front Psychol. 2023;14:1089570. 36891208 10.3389/fpsyg.2023.1089570 PMC9986600

[40] Kulikova A , Lopez J , Antony A , et al. Multivariate association of child depression and anxiety with asthma outcomes. J Allergy Clin Immunol Pract. 2021;9(6):2399‐2405. 33677079 10.1016/j.jaip.2021.02.043 PMC8195858

[41] Sun L , Ji S , Ye J . Canonical correlation analysis for multilabel classification: a least‐squares formulation, extensions, and analysis. IEEE Trans Pattern Anal Mach Intell. 2011;33(1):194‐200. 20733223 10.1109/TPAMI.2010.160

[42] Doherty C , Nowacki AS , Pat McAndrews M , et al. Predicting mood decline following temporal lobe epilepsy surgery in adults. Epilepsia. 2021;62(2):450‐459. 33464568 10.1111/epi.16800 PMC8216427

[43] Qiao J , Sui R , Zhang L , Wang J . Construction of a risk model associated with prognosis of post‐stroke depression based on magnetic resonance spectroscopy. Neuropsychiatr Dis Treat. 2020;16:1171‐1180. 32440132 10.2147/NDT.S245129 PMC7217706

[44] Fritz BA , Hoertel N , Lenze EJ , Jalali F , Reiersen AM . Association between antidepressant use and ED or hospital visits in outpatients with SARS‐CoV‐2. Transl Psychiatry. 2022;12(1):341. 35995770 10.1038/s41398-022-02109-3 PMC9395392

[45] Andreasen NC , Pressler M , Nopoulos P , Miller D , Ho BC . Antipsychotic dose equivalents and dose‐years: a standardized method for comparing exposure to different drugs. Biol Psychiatry. 2010;67(3):255‐262. 19897178 10.1016/j.biopsych.2009.08.040 PMC3677042

[46] Zhao Q , Yu CD , Wang R , et al. A multidimensional coding architecture of the vagal interoceptive system. Nature. 2022;603(7903):878‐884. 35296859 10.1038/s41586-022-04515-5 PMC8967724

[47] Quadt L , Critchley HD , Garfinkel SN . The neurobiology of interoception in health and disease. Ann N Y Acad Sci. 2018;1428(1):112‐128. 29974959 10.1111/nyas.13915

[48] Koban L , Gianaros PJ , Kober H , Wager TD . The self in context: brain systems linking mental and physical health. Nat Rev Neurosci. 2021;22:309‐322. 33790441 10.1038/s41583-021-00446-8 PMC8447265

[49] Weng HY , Feldman JL , Leggio L , Napadow V , Park J , Price CJ . Interventions and manipulations of interoception. Trends Neurosci. 2021;44(1):52‐62. 33378657 10.1016/j.tins.2020.09.010 PMC7805576

[50] van der Velden AM , Scholl J , Elmholdt EM , et al. Mindfulness training changes brain dynamics during depressive rumination: a randomized controlled trial. Biol Psychiatry. 2023;93(3):233‐242. 36328822 10.1016/j.biopsych.2022.06.038

[51] Datko M , Lutz J , Gawande R , et al. Increased insula response to interoceptive attention following mindfulness training is associated with increased body trusting among patients with depression. Psychiatry Res Neuroimaging. 2022;327:111559. 36308976 10.1016/j.pscychresns.2022.111559 PMC12981234

[52] Benasi G , Fava GA , Guidi J . Prodromal symptoms in depression: a systematic review. Psychother Psychosom. 2021;90(6):365‐372. 34350890 10.1159/000517953

[53] Dunne J , Flores M , Gawande R , Schuman‐Olivier Z . Losing trust in body sensations: interoceptive awareness and depression symptom severity among primary care patients. J Affect Disord. 2021;282:1210‐1219. 33601698 10.1016/j.jad.2020.12.092 PMC10398840

[54] Hermesdorf M , Berger K , Baune BT , Wellmann J , Ruscheweyh R , Wersching H . Pain sensitivity in patients with major depression: differential effect of pain sensitivity measures, somatic cofactors, and disease characteristics. J Pain. 2016;17(5):606‐616. 26867484 10.1016/j.jpain.2016.01.474

[55] Conejero I , Olié E , Calati R , Ducasse D , Courtet P . Psychological pain, depression, and suicide: recent evidences and future directions. Curr Psychiatry Rep. 2018;20(5):33. 29623441 10.1007/s11920-018-0893-z

[56] Staudacher HM , Mikocka‐Walus A , Ford AC . Common mental disorders in irritable bowel syndrome: pathophysiology, management, and considerations for future randomised controlled trials. Lancet Gastroenterol Hepatol. 2021;6(5):401‐410. 33587890 10.1016/S2468-1253(20)30363-0

[57] Sibelli A , Chalder T , Everitt H , Workman P , Windgassen S , Moss‐Morris R . A systematic review with meta‐analysis of the role of anxiety and depression in irritable bowel syndrome onset. Psychol Med. 2016;46(15):3065‐3080. 27605134 10.1017/S0033291716001987

[58] Tramullas M , Finger BC , Moloney RD , et al. Toll‐like receptor 4 regulates chronic stress‐induced visceral pain in mice. Biol Psychiatry. 2014;76(4):340‐348. 24331544 10.1016/j.biopsych.2013.11.004

[59] Wang XY , Xu X , Chen R , et al. The thalamic reticular nucleus‐lateral habenula circuit regulates depressive‐like behaviors in chronic stress and chronic pain. Cell Rep. 2023;42(10):113170. 37738124 10.1016/j.celrep.2023.113170

[60] Lyons N , Michaelsen MM , Graser J , Bundschuh‐Müller K , Esch T , Michalak J . Bodily experience in depression: using focusing as a new interview technique. Psychopathology. 2021;54(3):150‐158. 33951644 10.1159/000514128

[61] Dombrovski AY , Mulsant BH , Houck PR , et al. Residual symptoms and recurrence during maintenance treatment of late‐life depression. J Affect Disord. 2007;103(1–3):77‐82. 17321595 10.1016/j.jad.2007.01.020 PMC2680091

[62] Briggs JP , Shurtleff D . Acupuncture and the complex connections between the mind and the body. JAMA. 2017;317(24):2489‐2490. 28654992 10.1001/jama.2017.7214

[63] Jacob SN , Nienborg H . Monoaminergic neuromodulation of sensory processing. Front Neural Circuits. 2018;12:51. 30042662 10.3389/fncir.2018.00051 PMC6048220

[64] Hurley LM , Hall IC . Context‐dependent modulation of auditory processing by serotonin. Hear Res. 2011;279(1–2):74‐84. 21187135 10.1016/j.heares.2010.12.015 PMC3134116

[65] Broers C , Geeraerts A , Boecxstaens V , et al. The role of serotonin in the control of esophageal sensitivity assessed by multimodal stimulation in health. Neurogastroenterol Motil. 2021;33(3):e14057. 33280212 10.1111/nmo.14057

[66] Masdrakis VG , Markianos M , Baldwin DS . Apathy associated with antidepressant drugs: a systematic review. Acta Neuropsychiatr. 2023;35(4):189‐204. 36644883 10.1017/neu.2023.6

[67] McCabe C , Mishor Z , Cowen PJ , Harmer CJ . Diminished neural processing of aversive and rewarding stimuli during selective serotonin reuptake inhibitor treatment. Biol Psychiatry. 2010;67(5):439‐445. 20034615 10.1016/j.biopsych.2009.11.001 PMC2828549

[68] Murphy SE , Norbury R , O'Sullivan U , Cowen PJ , Harmer CJ . Effect of a single dose of citalopram on amygdala response to emotional faces. Br J Psychiatry. 2009;194(6):535‐540. 19478294 10.1192/bjp.bp.108.056093 PMC2802527

[69] Livermore JJA , Skora LI , Adamatzky K , Garfinkel SN , Critchley HD , Campbell‐Meiklejohn D . General and anxiety‐linked influences of acute serotonin reuptake inhibition on neural responses associated with attended visceral sensation. Transl Psychiatry. 2024;14(1):241. 38844469 10.1038/s41398-024-02971-3 PMC11156930

[70] Brown KW , Berry D , Eichel K , Beloborodova P , Rahrig H , Britton WB . Comparing impacts of meditation training in focused attention, open monitoring, and mindfulness‐based cognitive therapy on emotion reactivity and regulation: neural and subjective evidence from a dismantling study. Psychophysiology. 2022;59(7):e14024. 35182393 10.1111/psyp.14024 PMC9286350

[71] Dai J , Sun D , Li B , et al. Mixed‐mode mindfulness‐based cognitive therapy for psychological resilience, self esteem and stigma of patients with schizophrenia: a randomized controlled trial. BMC Psychiatry. 2024;24(1):179. 38439012 10.1186/s12888-024-05636-z PMC10913446

[72] Michalak J , Schultze M , Heidenreich T , Schramm E . A randomized controlled trial on the efficacy of mindfulness‐based cognitive therapy and a group version of cognitive behavioral analysis system of psychotherapy for chronically depressed patients. J Consult Clin Psychol. 2015;83(5):951‐963. 26371617 10.1037/ccp0000042

[73] Hofheinz C , Reder M , Michalak J . How specific is cognitive change? A randomized controlled trial comparing brief cognitive and mindfulness interventions for depression. Psychother Res. 2020;30(5):675‐691. 31694478 10.1080/10503307.2019.1685138

[74] Röhricht F , Papadopoulos N , Priebe S . An exploratory randomized controlled trial of body psychotherapy for patients with chronic depression. J Affect Disord. 2013;151(1):85‐91. 23769289 10.1016/j.jad.2013.05.056

[75] Winter D , Malighetti C , Cipolletta S , Ahmed S , Benson B , Röhricht F . Construing and body dissatisfaction in chronic depression: a study of body psychotherapy. Psychiatry Res. 2018;270:845‐851. 30551334 10.1016/j.psychres.2018.10.061

[76] Moyer CA , Rounds J , Hannum JW . A meta‐analysis of massage therapy research. Psychol Bull. 2004;130(1):3‐18. 14717648 10.1037/0033-2909.130.1.3

[77] Arnold MM , Müller‐Oerlinghausen B , Hemrich N , Bönsch D . Effects of psychoactive massage in outpatients with depressive disorders: a randomized controlled mixed‐methods study. Brain Sci. 2020;10(10):676. 32993175 10.3390/brainsci10100676 PMC7600300

[78] Craig AD . How do you feel? Interoception: the sense of the physiological condition of the body. Nat Rev Neurosci. 2002;3(8):655‐666. 12154366 10.1038/nrn894

[79] Zhou H , Zou H , Dai Z , et al. Interoception dysfunction contributes to the negative emotional bias in major depressive disorder. Front Psych. 2022;13:874859. 10.3389/fpsyt.2022.874859 PMC9035634 35479498

[80] Wang R , Chang RB . The coding logic of interoception. Annu Rev Physiol. 2024;86:301‐327. 38061018 10.1146/annurev-physiol-042222-023455 PMC11103614

