# Supplementary material for: Unveiling the interoception impairment in various major depressive disorder stages
Source: CNS Neurosci Ther. 2024 Aug 18;30(8):e14923. doi: 10.1111/cns.14923 (PMC11330652; doi:10.1111/cns.14923)
Supplement: Supplementary file 1 — Appendix S1 [file CNS-30-e14923-s001.docx]

**Measures of depression, anxiety and interception**

***Measures***

**Depressive symptoms**

The Patient Health Questionnaire-9 (PHQ-9) were used to evaluate the severity of depression. It is a well-validated tool developed based on DSM-5 criteria and encompasses the nine core symptoms that define depression.1 The PHQ-9 is a 9-item self-report depression screening tool for detecting major depressive disorder overall and by study or participant subgroups.^2^ This questionnaire comprises a 4-point ordinal response scale (from 0 = not at all to 3 = nearly every day) and higher scores indicating higher severity of symptoms. Following previous studies,^3^ the total PHQ-9 scores classified depressive state: no depressive symptoms (0 - 4); mild/subthreshold depression (5 - 9); and depression (≥ 10). The McDonald’s omega was 0.96 in the present study.

**Anxiety symptoms**

The Generalized Anxiety Disorder-7 questionnaire (GAD-7) was used to evaluate the severity of anxiety. GAD-7 is a 7-item self-report questionnaire to assess the presence and magnitude of generalized anxiety symptoms. It comprises a 4-point response scale ranging from 0 (not at all) to 3 (nearly every day). As with the PHQ-9, this questionnaire consists of a 4-point response scale ranging from 0 (not at all) to 3 (almost every day) and higher scores indicating higher severity of symptoms. A cutoff score of 5 was used to determine whether a participant had anxious symptoms. The McDonald’s omega was 0.94 in the present study.

**Interoception**

A Multidimensional Assessment of Interoceptive Awareness-version 2 (MAIA-2) was applied to assess interoception. The MAIA-2 is a 37-item self-report questionnaire containing eight continuous subscales: noticing (“I notice where in my body I am comfortable”), not distracting (“I distract myself from sensations of discomfort”), not worrying (“I can notice an unpleasant sensation without worrying”), attention regulation (“I am able to consciously focus on my body as a whole”), emotional awareness (“When something is wrong in my life, I can feel it in my body”), self-regulation (“When I am caught in my thoughts, I can calm my mind by focusing on my body/breathing”), body listening (“I listen to my body to inform me about what to do”), and trusting (“I trust my body sensations”). It evaluates a broad definition of interoception and can capture changes in multiple interoceptive awareness dimensions. The MAIA-2 is a diagnosis-independent measure and allows comparison across different illnesses.^4^ Higher scores indicate better self-reported interoception on a 6-point rating scale (0 = never, 5 = always). The Cronbach’s alpha was 0.71 in the current sample.

**Lifestyle and economic situation**

In addition to general demographic information (gender, age, BMI, education years), we also assessed the subjects’ lifestyle and economic context, including daily social media usage time, physical exercise, smoking and alcohol consumption, coffee and tea intake, marriage, childbearing, and total annual family income. All of the above factors have the potential to have an impact on interoception.^5^ These dimensions were evaluated through specific questions, such as “How much time do you spend on social media for entertainment?” Of them, the Physical Exercise Rating Scale (PARS-3) was used to assess the amount of exercise. It can evaluate the physical exercise of participants from three aspects: exercise intensity, frequency, and time. The formula “exercise intensity × exercise time × exercise frequency” was used to quantify the total score of exercise behavior, and the higher the score, the greater the exercise amount.

**2.3 Statistical Analysis**

We first compared the differences among three groups: healthy controls, subclinical group, and depression group. Subsequently, within the depression group, they further subdivided participants into first-episode, medication-naïve, and previously treated subgroups to analyze the effects of medication. This statistical method effectively reduces the number of comparisons, thereby lowering the Type I error rate.

**REFERENCES**

1. Malhi GS, Mann JJ. Depression. Lancet. 2018;392(10161):2299-2312.

2. Negeri ZF, Levis B, Sun Y, et al. Accuracy of the Patient Health Questionnaire-9 for screening to detect major depression: updated systematic review and individual participant data meta-analysis. *BMJ.* 2021; 375:n2183.

3. Sakata M, Toyomoto R, Yoshida K, et al. Components of smartphone cognitive-behavioural therapy for subthreshold depression among 1093 university students: a factorial trial. *Evid Based Ment Health.* 2022; 25(e1):e18-e25.

4. Heim N, Bobou M, Tanzer M, Jenkinson PM, Steinert C, Fotopoulou A. Psychological interventions for interoception in mental health disorders: A systematic review of randomised-controlled trials. *Psychiatry Clin Neurosci.* 2023; 77(10):530-540.

5. Morales-Torres R, Carrasco-Gubernatis C, Grasso-Cladera A, Cosmelli D, Parada FJ, Palacios-García I. Psychobiotic Effects on Anxiety Are Modulated by Lifestyle Behaviors: A Randomized Placebo-Controlled Trial on Healthy Adults. *Nutrients.* 2023;15(7):1706.
