# Supplementary material for: Unveiling the interoception impairment in various major depressive disorder stages
Source: CNS Neurosci Ther. 2024 Aug 18;30(8):e14923. doi: 10.1111/cns.14923 (PMC11330652; doi:10.1111/cns.14923)
Supplement: Supplementary file 2 — Appendix S2 [file CNS-30-e14923-s003.docx]

**The diagnostic efficacy of interoception for MDD**

Significant interoception variables obtained from the above analysis took part in the examination of diagnostic efficacy. The dataset of MDDs and HCs was randomly divided into training and validation cohorts at a ratio of 7:3, and the variables were compared by the Student’s *t*-test. The characteristics of interoception in training cohort and internal test cohort were shown in Table 1.

Table 1. The characteristics of interoception in training cohort and internal test cohort

| **Characteristics** | **Cohort** | | ***p*-value^2^** |
| --- | --- | --- | --- |
|  | **Training Cohort (N=640)** | **Internal Test Cohort (N=274)** |  |
| Noticing, mean (SD) | 2.58 (1.09) | 2.49 (1.17) | .236 |
| Not Noticing, mean (SD) | 2.94 (1.09) | 2.99 (1.05) | .522 |
| Not worrying, mean (SD) | 2.33 (0.90) | 2.34 (0.90) | .782 |
| Attention regulation, mean (SD) | 2.12 (1.00) | 2.07 (1.060 | .464 |
| Self-regulation, mean (SD) | 1.74 (1.21) | 1.76 (1.13) | .821 |
| Body listening, mean (SD) | 1.76 (1.24) | 1.74 (1.17) | .809 |
| Trusting, mean (SD) | 2.06 (1.25) | 2.05 (1.16) | .930 |

^1^Welch Two Sample t-test

The least absolute shrinkage and selection operator (LASSO) logistic regression analysis is a statistical method utilized for variable selection and regularization. It introduces a penalty term that shrinks certain coefficients to zero, thereby automatically selecting significant variables and effectively reducing model complexity. LASSO logistic regression can balance the accuracy and complexity of the model, preventing overfitting.^1^ In the training cohort, the least absolute shrinkage and selection operator (LASSO) logistic regression analysis was used for multivariate analysis to screen the independent risk factors. Eventually, 5 potential predictors (noticing, not distracting, not worrying, self-regulation, and Trusting), were included in the prediction model. The coefficients are shown in the following Table 2, and a coefficient profile is plotted in the following Figure 1. A cross-validated error plot of the LASSO regression model is also shown in Figure 2. The most regularized and parsimonious model, with a cross-validated error within one standard error of the minimum, included 5 variables.


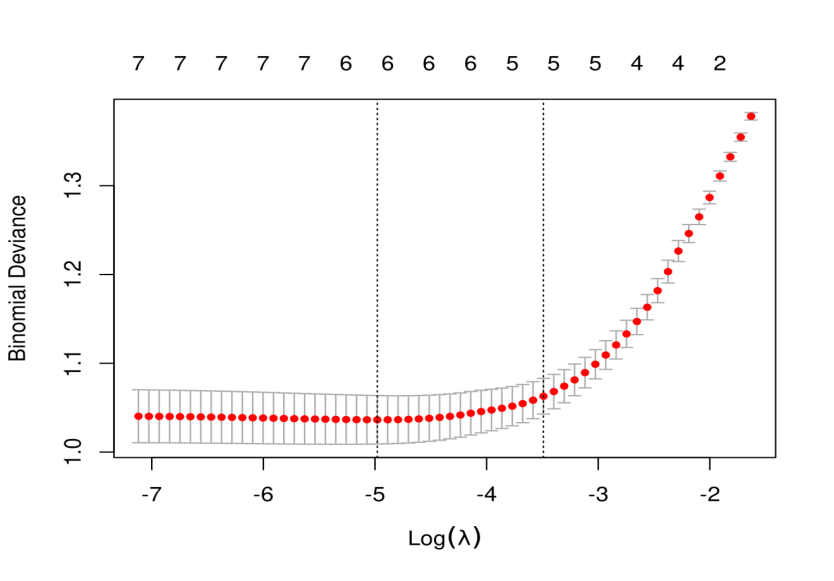


Fig.1 Lasso Regression Cross-Validation Plot


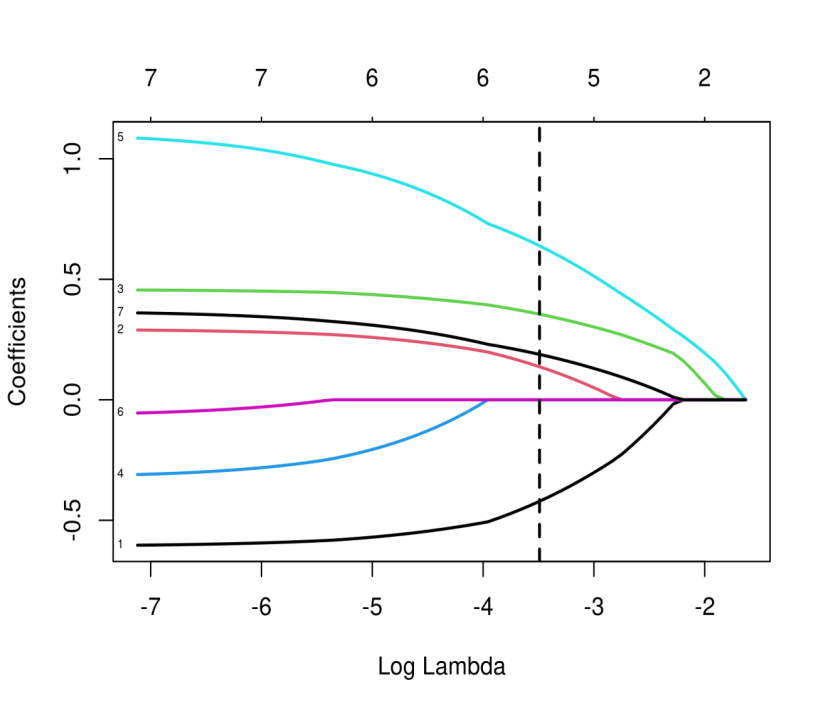


Fig. 2 Lasso Regression Coefficient Path Plot

Table 2. The coefficients of Lasso regression analysis

| **variable** | **Coefficient** |
| --- | --- |
| **(Interoception)** | -1.8484731 |
| Noticing level | -0.4211032 |
| Not.distracting level | 0.1370213 |
| Not.worrying level | 0.3552063 |
| Attention.regulation level | 0.0000000 |
| Self.regulation level | 0.6387818 |
| Body.listening level | 0.0000000 |
| Trusting level | 0.1882609 |

We also utilized a nomogram to visualize the predicted probabilities of MDD occurrence. A nomogram is a graphical tool that translates statistical models into a user-friendly format, enabling clinicians to estimate the risk of a specific event for individual patients. By quantifying the contributions of each predictive factor, the nomogram provides a practical and intuitive way for healthcare professionals to assess an individual’s risk of developing MDD.

The receiver operating characteristic (ROC) curve is a statistical tool used to evaluate the diagnostic performance of classification models. This curve illustrates the efficacy of a classifier at various decision thresholds by depicting the relationship between the True Positive Rate (TPR) and the False Positive Rate (FPR). TPR is defined as the ratio of the number of samples correctly classified as positive to the total number of actual positives, while FPR is the ratio of the number of negatives incorrectly classified as positives to the total number of actual negatives. These two metrics collectively reflect the classifier's ability to identify positive cases and distinguish negative cases from varying perspectives. The Area Under the Curve (AUC) of the ROC provides a means to quantify the model’s performance, with values ranging from 0.5 (no discrimination) to 1 (perfect discrimination). A high AUC value indicates that the classifier possesses a superior ability to distinguish between positive and negative cases.^2^ We applied the ROC analysis on 5 predictors. As shown in Figure 3, the ROC analysis of the 5 variables yielded AUC values greater than 0.5. Further multivariate logistic analyses were carried out in different cohorts. Results are shown in the following Table 3.


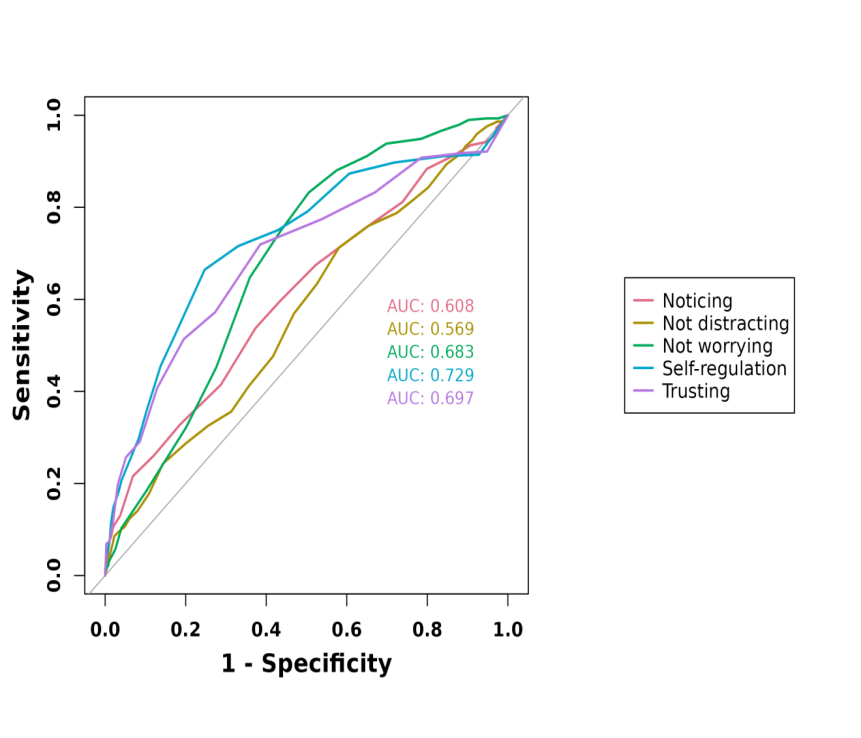


Fig. 3 ROC curve analysis of 5 candidate diagnostic indicators

Table 3. Results of Multivariate Logistic regression for Training Cohort

| **Characteristics** | **N** | **Event N** | **OR**^1^ | **95% CI**^1^ | ***p*-value** |
| --- | --- | --- | --- | --- | --- |
| Noticing | 640 | 292 | 0.50 | 0.39, 0.63 | < .001 |
| Not distracting | 640 | 292 | 1.39 | 1.13, 1.72 | .002 |
| Not worrying | 640 | 292 | 1.61 | 1.25, 2.09 | < .001 |
| Self-regulation | 640 | 292 | 2.55 | 2.00, 3.30 | < .001 |
| Trusting | 640 | 292 | 1.38 | 1.12, 1.70 | .002 |

^1^OR = Odds Ratio, CI = Confidence Interval

Decision Curve Analysis (DCA) is a method used in the evaluation of diagnostic tests, prediction models, and medical decision-making.^3^ It assesses the clinical utility of predictive models by considering the net benefit of different decision-making strategies across a range of threshold probabilities (i.e., the probability that a patient will choose that diagnostic option). It can compensate for the deficiency of ROC curves since it displays the false- and the true-positive fractions as functions of the risk threshold.^4^ In this study, DCA was performed to determine the net benefit threshold of prediction.

**REFERENCES**

**1.** Hu JY, Wang Y, Tong XM, Yang T. When to consider logistic LASSO regression in multivariate analysis? *Eur J Surg Oncol.* 2021; 47(8):2206.

**2.** Mandrekar JN. Receiver operating characteristic curve in diagnostic test assessment. *J Thorac Oncol.* 2010; 5(9):1315-6.

**3.** Fitzgerald M, Saville B, Lewis R. Decision Curve Analysis. *JAMA.* 2015; 313(4):409-410.

**4.** Kerr KF, Brown MD, Zhu K, Janes H. Assessing the Clinical Impact of Risk Prediction Models With Decision Curves: Guidance for Correct Interpretation and Appropriate Use. *J Clin Oncol.* 2016; 34(21):2534-2540.
