# Supplementary material for: Unveiling the interoception impairment in various major depressive disorder stages
Source: CNS Neurosci Ther. 2024 Aug 18;30(8):e14923. doi: 10.1111/cns.14923 (PMC11330652; doi:10.1111/cns.14923)
Supplement: Supplementary file 3 — Appendix S3 [file CNS-30-e14923-s002.docx]

**The demographics and baseline characteristics of MDDs with/without antidepressive therapy**

The demographics and baseline characteristics of MDDs without antidepressive therapy (MDD-A) and MDDs with antidepressive therapy (MDD+A), and antidepressive agents for MDD+A were listed in Table 1 and Table 2.

Table 1. MDD-A vs MDD+A demographics and baseline characteristics

| **Characteristics** | **Group** | | ***p*-value^2^** |
| --- | --- | --- | --- |
|  | **MDD-A (N=233^1^)** | **MDD+A (N=250^1^)** |  |
| Age, mean (SD), y | 26 (9) | 29 (11) | .008 |
| Gende, female, (%) | 160 (69%) | 155 (62%) | .124 |
| Education, mean (SD), y | 13.67 (2.94) | 12.88 (2.96) | .003 |
| Body Mass Index, mean (SD) | 21.8 (3.1) | 22.0 (3.5) | .423 |
| Physical exercise, mean (SD), hour/d | 10 (14) | 11 (16) | .564 |
| Disease duration, mean (SD), months | 20 (30) | 42 (53) | < .001 |
| Married, (%) |  |  | .444 |
| Married | 160 (69%) | 159 (64%) |  |
| Single | 64 (27%) | 82 (33%) |  |
| Divorced | 9 (4%) | 9 (4%) |  |
| Pregnancy, (%) |  |  | .287 |
| 0 | 189 (81%) | 188 (75%) |  |
| 1 | 37 (16%) | 48 (19%) |  |
| 2 | 6 (3%) | 13 (5%) |  |
| 3 | 1 (0%) | 1 (0%) |  |
| Frequency for cigarette smoking in the past month, (%) |  |  | .768 |
| No smoking | 195 (84%) | 205 (82%) |  |
| > 7 days | 8 (3%) | 8 (3%) |  |
| 2-7 days | 17 (7%) | 17 (7%) |  |
| Every day | 13 (6%) | 20 (8%) |  |
| Frequency for coffee and tea intake in the past month. (%) |  |  | .252 |
| No coffee and tea intake | 143 (61%) | 174 (70%) |  |
| > 7 days | 57 (24%) | 46 (18%) |  |
| 2-7 days | 22 (9%) | 22 (9%) |  |
| Every day | 11 (5%) | 8 (3%) |  |
| Frequency for alcohol drinkers in the past month, (%) |  |  | .850 |
| No drinking | 199 (85%) | 215 (86%) |  |
| > 7 days | 26 (11%) | 25 (10%) |  |
| 2-7 days | 1 (0%) | 3 (1%) |  |
| Every day | 7 (3%) | 7 (3%) |  |
| Daily social media usage time, hours, (%) |  |  | .383 |
| < 3 | 37 (16%) | 49 (20%) |  |
| 3-5 | 73 (31%) | 81 (32%) |  |
| 5-8 | 62 (27%) | 51 (20%) |  |
| >8 | 61 (26%) | 69 (28%) |  |
| Economic status (annual family income), Chinese Yuan, (%) |  |  | .010 |
| < 10,000 | 71 (30%) | 51 (20%) |  |
| 10000 - 30000 | 138 (59%) | 161 (64%) |  |
| 30000 - 50000 | 10 (4%) | 7 (3%) |  |
| > 50000 | 14 (6%) | 31 (12%) |  |
| Noticing, mean (SD) | 2.72 (1.04) | 2.76 (1.01) | .709 |
| Not distracting, mean (SD) | 2.69 (1.04) | 2.95 (1.03) | .005 |
| Not worrying, mean (SD) | 2.10 (0.87) | 1.99 (0.98) | .173 |
| Attention regulation, mean (SD) | 2.05 (0.94) | 1.94 (0.92) | .200 |
| Emotional awareness, mean (SD) | 2.43 (1.07) | 2.45 (1.17) | .823 |
| Self-regulation, mean (SD) | 1.46 (0.98) | 1.18 (0.92) | .001 |
| Body listening, mean (SD) | 1.66 (1.15) | 1.44 (1.08) | .035 |
| Trusting, mean (SD) | 1.72 (1.00) | 1.57 (0.95) | .091 |
| PHQ9, mean (SD) | 18.0 (5.1) | 20.4 (5.2) | < .001 |
| GAD7, mean (SD) | 12.5 (4.7) | 13.7 (4.8) | .007 |

^1^n (%); ^2^Welch Two Sample *t*-test; Pearson’s Chi-squared test; Fisher’s exact test

Table 2 The antidepressive agents for MDD+A (n = 250)

| Antidepressive agents | MDDs |
| --- | --- |
| Citalopram | 26 |
| Escitalopram | 22 |
| Duloxetine | 20 |
| Fluoxetine | 19 |
| Fluvoxamine | 18 |
| Mianserin | 18 |
| Mirtazapine | 12 |
| Paroxetine | 12 |
| Sertraline | 11 |
| Vortioxetine | 11 |
| Citalopram+Quetipine | 10 |
| Citalopram+Olanzapine | 9 |
| Fluoxetine+Quetipine | 14 |
| Fluoxetine+Olanzapine | 13 |
| Sertraline+Quetipine | 9 |
| Sertraline+Olanzapine | 8 |
| Duloxetine+Quetipine | 6 |
| Duloxetine+Olanzapine | 5 |
| Escitalopram+Quetipine | 4 |
| Escitalopram+Olanzapine | 3 |
